# Using standardized methods for research on HIV and injecting drug use in developing/transitional countries: case study from the WHO Drug Injection Study Phase II

**DOI:** 10.1186/1471-2458-6-54

**Published:** 2006-03-02

**Authors:** Don C Des Jarlais, Theresa E Perlis, Gerry V Stimson, Vladimir Poznyak

**Affiliations:** 1 Baron Edmond de Rothschild Chemical Dependency Institute, Beth Israel Medical Center, 160 Water Street, 24^th ^Floor, New York NY 10038, United States. Also at The Center for Drug Use and HIV Research, National Development and Research Institutes Inc., New York, NY 10010, USA; 2 Executive Director, International Harm Reduction Association. Also at the Centre for Research on Drugs and Health Behaviour, Imperial College of Medicine, St Dunstan's Road, London W6 8RP, UK; 3 World Health Organization, Management of Substance Dependence, CH-1211 Geneva 27, Switzerland

## Abstract

**Background:**

Successful cross-national research requires methods that are both standardized across sites and adaptable to local conditions. We report on the development and implementation of the methodology underlying the survey component of the WHO Drug Injection Study Phase II – a multi-site study of risk behavior and HIV seroprevalence among Injecting Drug Users (IDUs).

**Methods:**

Standardized operational guidelines were developed by the Survey Coordinating Center in collaboration with the WHO Project Officer and participating site Investigators. Throughout the duration of the study, survey implementation at the local level was monitored by the Coordinating Center. Surveys were conducted in 12 different cities. Prior rapid assessment conducted in 10 cities provided insight into local context and guided survey implementation. Where possible, subjects were recruited both from drug abuse treatment centers and via street outreach. While emphasis was on IDUs, non-injectors were also recruited in cities with substantial non-injecting use of injectable drugs. A structured interview and HIV counseling/testing were administered.

**Results:**

Over 5,000 subjects were recruited. Subjects were recruited from both drug treatment and street outreach in 10 cities. Non-injectors were recruited in nine cities. Prior rapid assessment identified suitable recruitment areas, reduced drug users' distrust of survey staff, and revealed site-specific risk behaviors. Centralized survey coordination facilitated local questionnaire modification within a core structure, standardized data collection protocols, uniform database structure, and cross-site analyses. Major site-specific problems included: questionnaire translation difficulties; locating affordable HIV-testing facilities; recruitment from drug treatment due to limited/selective treatment infrastructure; access to specific sub-groups of drug users in the community, particularly females or higher income groups; security problems for users and interviewers, hostility from local drug dealers; and interference by local service providers.

**Conclusion:**

Rapid assessment proved invaluable in paving the way for the survey. Central coordination of data collection is crucial. While fully standardized methods may be a research ideal, local circumstances may require substantial adaptation of the methods to achieve meaningful local representation. Allowance for understanding of local context may increase rather than decrease the generalizability of the data.

## Background

While the injection of illicit psychoactive drugs has traditionally been associated with industrialized countries, particularly the US [1], the problem is increasing rapidly in developing and transitional countries. The most recent estimate of the numbers of injecting drug users (IDUs) found that 10 million of the 13 million estimated IDUs live in developing and transitional countries [2]. Injecting drug use can create multiple serious health and social problems in developing and transitional countries. The spread of HIV through the multi-person use (sharing) of needles and syringes for drug injecting is among the most dramatic and potentially catastrophic of these problems [3].

Rapid implementation of HIV prevention programs for IDUs is needed to address the problem of HIV transmission among IDUs in developing/transitional countries. Additional research is also needed in order to assess the specific prevention needs, to adapt existing programs to the local situation, and to assess the effectiveness of the implemented programs. Further research is also needed to develop generalizable knowledge that could be used to increase the effective use of the scarce resources available for preventing HIV infection among IDUs in developing and transitional countries.

Conducting research on HIV infection and HIV risk behaviors among IDUs in developing and transitional countries involves overcoming many difficulties, from contacting "hard to reach" populations to a limited research infrastructure. The use of "standardized" research methods across different sites is one of the more powerful tools for developing generalizable knowledge in epidemiology. The first WHO Multi-site Study of HIV and Injecting Drug Use (WHO Phase I), conducted from 1989 through 1993 [4], was one of the first studies that utilized standardized methods for collecting data in both resource-rich and resource-constrained countries, though the majority of sites were in resource-rich countries. This study produced a number of important findings with respect to preventing HIV infection among IDUs, including: 1) that IDUs were capable of changing their behavior to reduce the risk of HIV infection, and this behavior change had a strong protective effect against becoming infected [5], and 2) if effective prevention programming was implemented early and on a large scale, it was possible to avert epidemics of HIV among IDUs [6]. However, one of the major drawbacks of the study was the lack of qualitative contextual assessment.

The purpose of this paper is to report on the survey research methods used in the second phase of the World Health Organization Multi-Site Study of HIV/AIDS and Injecting Drug Use (WHO Drug Injection Study Phase II). This has been the largest study to utilize standardized methods to investigate HIV infection and risk behaviors among IDUs in developing and transitional countries. We wish to provide a reasonably detailed description of the methods in order that 1) persons examining data from the study will have a resource for understanding how the data were collected, 2) other researchers may utilize (and adapt) these methods for use in new studies, and 3) to illustrate some of the complex issues in using standardized methods across a wide variety of international sites.

## Methods

The WHO Drug Injection Study Phase II was undertaken from 2000 to 2004, and extended the aims of WHO Phase I to include study of transition from non-injecting to injecting drug use, investigation of overdose and other adverse health consequences of drug use, and estimation of hepatitis B and C prevalence among injecting drug users. This second study was specifically designed to incorporate a rapid assessment and response (RAR) component in addition to a cross-sectional seroprevalence and risk behavior survey of injecting drug users. The purpose of RAR was to implement methods for speedy research to keep pace with the rapid spread of HIV, to inform further in-depth studies where needed, and to encourage early intervention. (A technical guide to RAR can be found on the web [7].) The subsequent survey component, while designed to be similar to the survey conducted in Phase I, thus had the advantage of being able to draw on information obtained during the RAR. In contrast to WHO Phase I, the Phase II study was designed to be conducted solely in resource-constrained countries.

Twelve cities participated in the WHO Drug Injection Study Phase II survey including two, Rio de Janeiro and Santos in Brazil, that were also part of WHO Phase I. New cities included Lagos (Nigeria) and Nairobi (Kenya) in Africa, Beijing (China), Hanoi (Vietnam), and Penang (Malaysia) in Asia, Kharkiv (Ukraine), Minsk (Belarus) and St. Petersburg (Russia) in Eastern Europe, Bogotá (Colombia) and Gran Rosario (Argentina) in South America. (Madras in India was also selected to participate but withdrew early in the study. Tehran in Iran joined the project approximately a year after the other cities, but had completed only the rapid assessment component at the time of writing.)

### Study coordination

Overall project management was carried out by the Department of Mental Health and Substance Dependence, WHO (WHO/MSB). Additionally, two coordinating Centers were established to work in cooperation with the WHO/MSB Study Team. The Centre for Research on Drugs and Health Behaviour at Imperial College School of Medicine in London was responsible for the RAR component and the NYC Survey Coordinating Center (NYC-SCC) of National Development and Research Institutes, Inc., and The Baron Edmond de Rothschild Chemical Dependency Institute, Beth Israel Medical Center in New York City was responsible for the multi-city questionnaire and seroprevalence survey. (Detailed information concerning the implementation of the rapid assessment component in the study has been described elsewhere[8,9].)

### Participating site selection

Based on recommendations from the Phase I study for future research to include representation from Africa, Eastern Europe, Latin and South America, the Caribbean, and parts of Asia in order to study a broader range of cultural environments, proposals were solicited from research institutions or agencies in these geographic areas. Potential participants were selected by the WHO/MSB team based on the scope of the local HIV epidemic among IDUs, and the presence of experienced investigators with access to resources to undertake both the RAR and the questionnaire and seroprevalence survey. Proposal guidelines issued by WHO/MSB covered the following: background and rationale for inclusion of the site in the study; qualifications of the investigating team and implementing agency; aim, scope, and duration of the project; plans for RAR and survey implementation, evaluation, and monitoring; expected project outputs and plans for utilization; partners and alliances; resources and budget; and procedures for local ethical clearance.

### Planning meeting

In September, 2000, after selection of most of the participating sites, a 4-day planning meeting was held in St. Petersburg, Russia. This was attended by the WHO/MSB project team, the Coordinating Center teams, and representatives from all participating cities except Nairobi which had not yet been selected at that time. The meeting provided an opportunity for training in all aspects of the survey and for resolution of problems and modifications to the questionnaire and survey process based on feedback from the investigators. Additional training in the rapid assessment and survey components was subsequently organized for the three East European research teams.

### Local partners and alliances

A wide variety of organizations and individuals collaborated with the Implementing Agencies and the Principal Investigators in planning and conducting the survey. Arrangements were made by each participating site individually. Limited funding was supplied by WHO/MSB, supplemented by additional funds obtained locally. Advisory committees, technical expertise in the areas of survey implementation, serology testing and statistical analysis, and assistance with identification of recruitment locations and fieldwork were drawn from academic institutions, medical facilities, local or national government authorities, non-governmental organizations, law enforcement units, and drug users.

### Responsibilities of the NYC survey coordinating center

In the Phase I study, despite the standardized survey methodology developed specifically for the study, lack of centralized coordination resulted in unilateral adaptation of survey procedures and difficulties in merging the datasets, thus limiting the potential for comparative analyses. The aim in Phase II was to build on and adapt the Phase I protocol to ensure adherence to standardized procedures and uniformity of data collection, while providing maximum flexibility to individual participating sites to adapt the protocol to local conditions.

Duties of the NYC-SCC included:

1. Review of proposals from prospective investigators.

2. Development of the overall survey protocol from Phase I and preparation of operational guidelines for sampling, recruitment, interviewing using a structured questionnaire, HIV testing and counseling, and data management. (A copy of the Operations Manual is available on the WHO project website [10].)

3. Revision of the WHO Drug Injecting Study Phase I survey questionnaire, incorporating suggestions from WHO/MSB and from potential WHO study collaborators or consultants, to create a modified questionnaire prototype with flexibility for local adaptation.

4. Organization of training workshops in Survey Methods.

5. Provision of technical support to participating sites in a) adaptation of the prototype questionnaire for local use; b) development of local study protocols, while ensuring their consistency with the overall study protocol.

6. Supervision of sampling, quality control, data collection and registration, ensuring cross-city compatibility.

7. Compilation and checking of data from the participating sites, and maintenance of the central dataset

8. Analysis of multi-site data.

### Responsibilities of participating sites

Following guidelines issued by the NYC-SCC and utilizing information and experience gained during the rapid assessment, each site was required to plan the local survey operations, adapt and translate the prototype questionnaire (prepared by NYC-SCC team) for local use, hire and train fieldworkers, pilot the questionnaire, identify recruitment locations, develop local sampling/recruitment protocols, recruit and interview subjects, select laboratory facilities and arrange for HIV serotesting of all interviewed subjects, enter questionnaire data into a computer database for forwarding to the NYC-SCC, and prepare interim and final reports on the survey.

### Ethical procedures

Principal Investigators and the Implementing Agencies were required to be familiar with the International Guidelines for Ethical Review of Epidemiological Studies [11], and to adhere to the principles described therein. Each participating site was required to submit their study protocol for review by a local Ethics Board composed of local scientists, researchers, representatives of NGOs, and others, and to prepare an informed consent information sheet to show (or read) to subjects to ensure that they were made aware of possible implications of participation in the research. The operational guidelines specified that the consent form should include: i) the right to refuse participation; ii) the right to withdraw from participation at any time without adverse consequences; iii) a statement emphasizing that participation was voluntary; iv) specification of a contact person to discuss/explain details of project; and v) assertion of confidentiality. Unless the local Ethics Board recommended that oral consent would be preferable for maintaining confidentiality, written consent was required of study recruits.

### Overall survey protocol and design

The primary focus of the study was on injecting drug use, thus each participating site was asked to try to recruit 400 current injectors, with a minimum of 100. The target of 400 was based on experience from WHO Phase I. The locally-adapted structured questionnaire was administered by a trained interviewer to each eligible recruit. Subsequently each subject was asked to provide a blood sample for HIV (and in some cases HBV and HCV) testing.

Although drug treatment programs provide a readily accessible pool of potential recruits for a drug study, the restrictive admittance criteria (e.g., high fees, HIV negative status) employed by some programs limit treatment to specific subgroups of drug users. Moreover, many drug users do not seek treatment. Thus in order to obtain broad coverage of injectors and reduce the bias associated with recruitment from treatment programs only, it was recommended that recruitment be carried out both from treatment program settings and from community or other non-treatment settings. The purpose of including subjects from both treatment and community settings was not to compare drug users entering treatment with those who did not seek, or lacked access to, treatment, but rather to provide findings that were generalizable across a broad range of IDUs. However, it should be noted that although comparison of subjects recruited from each source was not an analytic focus at the global level, such comparisons do provide a methodological check of bias at the city level.

In order to effectively target subsequent interventions, examination of reasons for transition from non-injecting to injecting drug use and understanding of reasons for difference in prevalence levels of drug injecting behavior in similar and geographically close cities was needed. Thus, in geographic areas where injection drug use was on the increase or where use of "injectable" drugs by non-injected modes of administration was widespread, individual sites were encouraged to also recruit persons who had replaced injection drug use with non-injection (former injectors), and/or persons who had never injected (never-injectors) in the study. These persons could be recruited from treatment and/or community settings as desired, however, a minimum of 100 former or 100 never-injectors was recommended.

### Eligibility for survey enrollment

Since most of the questionnaire sections on drug use and risk behaviors covered the 6-month period preceding study recruitment, it was decided to limit recruitment from treatment settings to clients newly-admitted to a treatment program within the past 30 days, since such clients could be considered to have been "part of the community" for most of the six-month period. Accordingly the questionnaire items would apply equally to both community and treatment recruits. Apart from the current course of treatment, the subject should not have been in that same treatment program or any other treatment program during the preceding 6 months. Community recruits could be drawn from any non-treatment settings, preferably from street locations. If a person recruited from a community setting was currently attending drug treatment this did not render him/her ineligible as a community recruit.

All sites were required to recruit current injectors, but recruitment of former or never-injectors was optional depending upon the local situation. The criteria for each category were:

a) Current injectors must have injected during the last 2 months

b) Former injectors had to have some history of injection but no injected drug use during the preceding 6 months in order to demonstrate a serious commitment to injection cessation. However they had to have used non-injection methods for "injectable" drugs during the preceding 2 months.

c) Never-injectors were persons who had never injected in their lifetime. However, they had to have used non-injection methods for "injectable" drugs during the preceding 2 months.

Finally, no subject could be recruited into the study more than once.

These selection criteria aimed at ensuring comparability across cities in different cultural environments and at different time periods. The term "injectable" was originally conceived to include drugs such as heroin, cocaine, and other commonly injected drugs. However, during rapid assessment the Bogotá team encountered instances of alcohol injection; from then on "injectable" was defined to cover any substance that anyone injected outside of a medical context.

### Selection and training of field staff

Field staff were drawn from a variety of backgrounds, from health-care professionals to NGO staff to recovering drug-dependent persons. Training for field workers was conducted following guidelines prepared by the NYC-SCC, although each participating site assumed responsibility for the precise nature and duration of the training sessions. Typically training lasted between two and five days and covered all aspects of recruitment and questionnaire administration, informed consent procedures, principles of communication with IDUs, safety issues for field workers, record keeping, and data management.

### Sampling procedures and recruitment of subjects

Owing to the vastly different situations across cities, a uniform approach to sampling and recruitment was not considered feasible. Overall guidelines were provided, but the ultimate decision on design and implementation of local sampling and recruitment protocols was made by each site investigator in consultation with the Survey Coordinating Centre.

#### Selection of treatment programs

During the rapid assessment investigators identified treatment programs or other institutions providing drug treatment (such as psychiatric institutions) and gathered data on service capability, patient turnover and "typical" patients. This information was used to determine whether recruitment from treatment programs was feasible and, if so, how many and which treatment programs should be included in the survey in order to provide the required sample size. It was also necessary to ensure that treatment staff would cooperate, or, in some instances, actually help to recruit. In most cities, treatment programs were selected based on size of the intake population and the ease of access to the target groups. Since it was important to avoid any selection bias on the part of the investigators, data collectors, or subjects, the project guidelines indicated that, if possible, subjects should be randomly selected from all newly admitted patients. Where random selection was not feasible (due primarily to small numbers of incoming patients), sequential new admits were screened for eligibility and all meeting the criteria were invited to participate in the survey.

#### Identification of community locations

Rapid assessment findings from in-depth interviews, focus groups, informal discussions, direct observation in natural settings, anonymous self-administered questionnaires, and geographic mapping were used to target locations most likely to yield potential subjects. In most cities repressive local policies about drug use and stigmatization of HIV-infected persons resulted in IDUs being a "hidden" population. Since random selection of subjects in the community was not feasible, techniques such as peer-referral, snowball sampling, and targeted sampling, were used in order to access the hidden populations. Investigators were instructed to make every effort to obtain a sample representative of the non-treatment population in the area.

### Questionnaire

#### Design

In order to maintain a high degree of continuity with the questionnaire from the first Drug Injecting Study and permit temporal comparisons in cities which participated in both phases, some sections or items from the Phase I questionnaire were duplicated in the questionnaire prototype prepared for the second study. However, to make space for new material, some previous sections had to be eliminated or reduced in length in order to avoid a prohibitively long new questionnaire. Sections in the modified questionnaire were classified as either "core" (to be included in questionnaires at all participating sites) or "local option" (to be included or excluded at the discretion of the local investigator).

#### Review process

Initial drafts of the revised questionnaire were sent to the WHO/MSB Project Officer, Principal Investigators from WHO Phase I, and other researchers throughout the world. Comments and suggestions were received from collaborators or consultants in Brazil, China, Colombia, Denmark, Greece, Italy, Netherlands, Nigeria, Russian Federation, Spain, Thailand, Ukraine, United Kingdom, and the United States. Subsequent drafts were reviewed in a WHO meeting in London and were also selectively pre-tested in New York and Bangkok. Reviewers' comments covered the spectrum, including: too long; not enough detail; use three different questionnaires (one for current injectors, one for former injectors, and one for never-injectors); split the questionnaire into one short section containing essential questions, and one longer section to administer to subjects willing to spend the time; place a greater focus on global diversity in substances used, behaviors, environments; and provide for the inclusion of questions targeting local behaviors or activities. An attempt was made to try to incorporate all suggestions made by at least two reviewers and to allow for more local modification and additions. Site-specific flexibility was built into many questions so that additional items could be added to multi-item questions, or additional response categories added to individual items. A penultimate version of the Phase II questionnaire was field-tested in Rio de Janeiro and Santos.

#### Final questionnaire

Core sections included demographics, injection drug use and associated behaviors, sexual behaviors, travel, HIV and AIDS awareness and behavior change, medical history, service utilization, witnessed overdose, and HIV testing prior to interview. Local option sections covered non-injecting drug use, injection initiation, last injection event, drug roles, hepatitis, experienced overdose, and involvement in violence. (A copy of the questionnaire may be obtained from the primary author, Dr. Des Jarlais, or the second author, Dr. Perlis.)

#### Local adaptation

The Principal Investigator at each participating site was responsible for adapting the questionnaire to reflect local circumstances by adding new items or questions or expanding the response range. Technical support was provided by the NYC-SCC team to ensure consistency with the research protocol for the whole study. Local modifications to the questionnaire were based partially on extant knowledge and partially on information collected during the rapid assessment stage. For example, categories of drug treatment and types of drugs used varied substantially across geographic regions. Moreover, in some cities, the presence of repressive law enforcement policies resulted in local risk behaviors not observed elsewhere.

#### Translation

Translation of the questionnaire was carried out locally at each site, – into Mandarin in Beijing, Vietnamese in Hanoi, Malay in Penang, Spanish in Bogotá and Rosario, and Portuguese in Rio de Janeiro and Santos. The three Eastern European countries collaborated on translation into Russian. Unfortunately, limited resources precluded back-translation. In Nairobi and Lagos the English version was used, but in the latter city interviewers interpreted as needed, due to the numerous different languages used in that area.

### Seroprevalence component

Serum samples were collected in all participating cities except Penang. The remaining eleven participating cities conducted testing for HIV, hepatitis B, and hepatitis C.

### Centralized data coordination

In order to ensure comparability of data across sites, all locally adapted questionnaires were required to conform to a uniform question numbering structure. A data entry system for the questionnaire prototype was designed by NYC-SCC using Epi Info™ version 6.04, and customized for each site that wanted to use Epi Info™ for its locally modified questionnaire For other sites preferring to set up their own data entry system (in SPSS^® ^or SAS^®^), a protocol specifying variable names, variable types, and data file formats was issued. Each site was asked to submit a small pilot data set (typically 10 cases) for checking and approval by the NYC-SCC before data-entering the full set of questionnaire data. When final complete data sets were submitted, NYC-SCC performed validity checking, requested corrections where necessary, then merged all datasets into one master dataset.

### Utility of RAR in planning and carrying out the survey

During the rapid assessment information was gathered from a wide variety of informal sources, including IDUs, other drug users, members of IDUs' social networks, drug dealers, non-users in contact with users in dealing and drug-using locations, students (high school and technical school), and formal sources including education system representatives, drug treatment specialists/agencies, health promotion programs and outreach teams, local health services, hospital emergency rooms, community leaders, law enforcement personnel, and local/national government officials. Modes of data collection included structured interviews using brief questionnaires, semi-structured interviews, direct observation in the field, informal interviews, focus groups, secondary data analysis, and content analysis of media publications. In individual cities the rapid assessment became an important tool in planning survey procedures such as recruitment and interviewing at the local level, modifying the questionnaire to reflect the local situation, and training of survey field staff.

## Results

### Overall recruitment

A total of 3350 current injectors and 1844 former or never-injectors meeting eligibility criteria were recruited (Tables 1a and 1b). Ten cities recruited from both treatment and community settings, however only Hanoi and the three Eastern European cities were able to reach the proposed recruitment goal of approximately 200 current injectors from each of the settings. Former injectors were recruited in nine cities (although the sample size in Beijing was very small), and never-injectors in seven cities. However, in some cities such recruitment occurred unintentionally due to lack of adherence to recruitment criteria resulting in erroneous recruitment of "current" IDUs who were later reclassified as former or never injectors. Additionally some recruits reported their last injection as being between 3 and 6 months prior to recruitment, thus failing to meet the study criteria for current or for former injectors, and had to be dropped from the analyses. (These persons are not included in the totals shown above.) In the South American cities cocaine was the typical drug of abuse, whereas heroin or other opiates were the primary drugs in all of the other cities except for Lagos where both heroin and cocaine featured prominently.

### Treatment recruitment

During the rapid assessment it became apparent that Lagos and Bogotá would not be able to recruit any current IDUs from treatment programs. In Lagos there were very few treatment opportunities and these were reserved for the wealthy. In Bogotá, although a prior local survey had identified over 82 drug treatment programs, analysis of the WHO rapid assessment data indicated that most persons admitted to drug treatment did not have a history of injection and only 2% to 3% of new admittances reported having tried injection even once or twice. The Bogotá team thus changed their survey plan to conduct community recruitment only. Similarly, rapid assessment in Rosario revealed a poor treatment infrastructure and evidence that injectors were typically not in treatment, partially due to the abstinence-based focus of most treatment programs. However, the Rosario team decided to attempt treatment recruitment by including all area treatment programs with any record of attendance by IDUs.

Although selection of treatment programs for inclusion in the study was primarily based on size of patient population, the type of treatment institution varied by site (Table 2). In the Asian countries, where drug use is highly criminalized, most study subjects were recruited from the large compulsory long-term rehabilitation programs. In Eastern Europe (where only current injectors were recruited) free drug treatment requires that the patient be officially registered as a person with a drug use disorder – thus many users prefer to pay for private treatment or to avoid treatment altogether.

Recruitment was typically carried out by study research staff, with assistance from program staff in some cities. Once the survey was underway, recruitment of current IDUs proved more difficult than anticipated from treatment programs in Nairobi, Penang, and the South American cities. In Nairobi, an attempt to recruit subjects from treatment settings yielded low turnout. Addicts entering the rehabilitation programs in Penang are typically mandated by the court to undergo rehabilitation at the program for 18–24 months, thus the turnover rate is rather slow and study recruitment was low. In the South American cities drug treatment was both relatively scarce, and primarily available only for non-injecting drug users, limiting recruitment possibilities.

### Community recruitment

Recruitment from community settings proved more difficult than anticipated in Lagos, Nairobi, Beijing, Penang, and Santos. In many cities key informants or "gatekeepers" who had assisted with the rapid assessment stage continued to assist with survey recruitment. Even in cities where the survey utilized new field staff, the carryover of the study "presence" from the rapid assessment stage facilitated survey recruitment greatly. In some cities, monetary incentives (Beijing, Lagos, Nairobi) or transportation expenses (Bogotá, Rio) were provided. The snowball sampling technique was typically used to recruit study subjects.

In most cities, many drug users came originally from middle (or even upper) income families. Except in the city of Lagos, almost all study subjects lived in relatively stable housing (their own or with family or friends), and approximately 50% or more of the study subjects in all cities engaged in some form of permanent or temporary work. Based on individual site reports, a brief description of community recruitment is provided for each site separately.

#### Lagos

Four of the twenty local government areas in Lagos State were selected based on known high level of drug activities, including the presence of a considerable number of drug selling points (so-called "joints") and drug users. This was the only city in the study in which large numbers of the users were homeless (living in the "joints", uncompleted buildings, in the market or other outdoor places), existing on money from temporary jobs or begging.

#### Nairobi

Similar to the approach in Lagos, four residential areas were selected based on high activity of drug use and sales: the City Center (located in the business district), Eastlands (a mix of low to middle class residential and business areas), Westlands (a mix of low-income and affluent groups), and peri-urban locations where the majority of heroin users were drivers and touts operating mini buses to and from the city center. In Nairobi most drug users worked to support themselves, and almost three quarters of the Nairobi recruits were engaged in some form of work, primarily temporary jobs.

#### Beijing

Rapid assessment had found that many drug users were small or large scale business owners selling a diverse range of goods and services including, travel, clothing, cigarettes, property, marketing, cotton and hay, computers, and mobile phones. Community recruitment of drug users proved particularly difficult in Beijing – only 50 recruits over several months of effort. Rapid assessment revealed that drug users frequently prefer to use in the privacy of their own homes (to reduce the risk of arrest), and that many injectors prefer to inject alone. Furthermore the huge travel distances within Beijing city hindered access to existing drug user networks. Thus peer and key informant referral (particularly drug dealers) proved to be a more successful strategy than snowball sampling. Regrettably, key informants, potential recruiters and subjects all feared arrest, inhibiting study recruitment. Moreover, many drug users appeared to be relatively wealthy and therefore not interested in the monetary incentive offered for study participation. Indeed, over a quarter of the recruits reported that they were living on savings.

#### Hanoi

Cluster sampling of persons from communes within districts with high numbers of drug users (according to the official registry of drug users maintained by the Provincial Sub-Departments for Social Evils Control) was used to ensure geographic coverage of the city. Initially five of the highest drug-use urban districts were selected, followed by selection of five of the highest drug-use communes in each district. Within each commune, an initial drug user was chosen by the peer educator from the local HIV/AIDS program, with subsequent drug users selected by snowball sampling. While many drug users worked at temporary jobs or family businesses, almost one third of community recruits were primarily supported by friends or relatives.

#### Penang

The selection of specific recruitment areas was based on information provided by the police and the National Narcotics Agency. One such area was a fishing village, where it was believed that drugs were easily obtained via the sea. Unlike the other cities, practically all drug users were working (many were fishermen) and none of the study recruits were supported by friends or family. Over 90% of the recruits classified themselves as middle or upper class. Official statistics in Malaysia indicate that female drug users are very rare, and none were actually recruited for the study.

#### Kharkiv

According to rapid assessment reports, IDUs are to be found in all areas of the city, with concentrations in areas around student and workers' hostels. The survey covered four districts in the city where drug injection activity was known to be high: Moskovskiy (the large central district), Frunzenskiy (industrial and poor), Kominternovskiy (mixed, containing a number of high educational institutions and hostels), and Dzerdzinskiy (mixed, located at the crossing of different districts, containing a number of high educational institutions and hostels). Both registered and non-registered IDUs were targeted. A large number of subjects (80%) reported some form of employment, although much of this was unskilled or temporary work.

#### Minsk

Initially, the Minsk research team obtained epidemiological data for all districts on overdose rates, reported number of drug-dependent persons and current rate of change, HIV and HBV infections, and drug trafficking crimes, and used these data to rate districts by high, average, or low levels of drug addiction. However, rapid assessment indicated that injection drug use occurred in all districts of the city, and that risk behaviors did not vary by district, thus the sample could be drawn from any or all districts. Clients from the National Center for AIDS Prevention, the Syringe Exchange, the Narcotics Anonymous group, and the NGO Positive Movement assisted with snowball recruitment in the streets, in discos and other public places.

#### St. Petersburg

Rapid assessment indicated that most IDUs live in the new districts of St. Petersburg (so called "bed-districts"), and IDUs congregate at metro stations, so it was decided to recruit the community subjects mainly in those locations. The old centre of St. Petersburg was the other region for recruitment. The research field staff drove to those locations in a van so that study recruits could be interviewed inside the vehicle and be less visible to police or other citizens.

#### Bogotá

During rapid assessment, drug injection practices were most visible among a few specific sub-groups – informal workers, handicraft and jewelry vendors who were typically involved in drug-dealing or other illegal activities. Recruitment from these groups was relatively easy due to their accessibility. However, there was also evidence that drug injecting was spreading through diverse social groups throughout the city, thus snowballing and peer referral strategies were implemented to access new groups. Additionally, the study team distributed leaflets asking for volunteers for the project. Recruits included grade and high school students, university students, workers in the informal and formal sectors, handicraft and jewelry vendors, athletes, drug dealers, people involved in other illicit activities and young people without any known occupation. Most subjects belonged to low and middle-income social levels, and a large number (43%) were supported by friends or family.

#### Gran Rosario

The research team identified public places where large numbers of discarded syringes were found and areas where drug users congregated. Study subjects were recruited from a variety of socio-economic groups, persons with permanent employment, temporary work, on government subsidies, persons supported by friends or family, persons involved in illegal activities, and street peddlers.

#### Rio de Janeiro

Drug use appears in all strata of society. Outreach workers were able to recruit drug users from many different communities including both middle class neighborhoods and economically disadvantaged communities. However, it was clear to the research team that drug users from the upper classes were considerably undersampled. A van was used to facilitate travel to different areas, and transportation expenses were paid to drug users where necessary. 80% of study recruits supported themselves primarily by income from work.

#### Santos

As in Rio, drug use is found among all socio-economic groups, however study subjects were mainly recruited from the lower and middle classes, including the city's tenements and slums.

### Demographics of study subjects

Tables 3a and 3b present the gender and age breakdown of all recruits in each of the cities. Females typically comprise less than one quarter of the overall sample in each city; indeed in Lagos, Nairobi, and Rio de Janeiro very few females were recruited and in Penang there were none. The proportion of females does not differ much between the current, former, or never-injectors except in Nairobi and Hanoi where the proportion of females is higher among current IDUs than among the other recruits. These two cities are also the two cities with the highest percentage of female sex workers in the IDU sample. In Hanoi all except one of the never-injectors are male.

Within each city, average age is similar for current, former, and never-injectors except that never-injectors in Penang and Santos and current IDUs in Bogotá and Rio de Janeiro tend to be younger than the other recruits. However, there are large age differences between sites – the Eastern Europe and Bogotá recruits tended to be the youngest, and the Lagos and Penang recruits the oldest. Indeed, in Bogotá 43% are under 21, and almost a third of Penang recruits are over 40.

In most cities, drug users on the whole are typically members of the ethnic majority groups. However, in Penang current and former IDUs are far more likely to be from the minority Chinese and Indian ethnic groups than the never-IDUs who are primarily Malay. Similarly, in Rosario current and former IDUs are more likely to be from the minority Mestizo or indigenous groups than the never-injectors who are primarily White.

## Discussion

The WHO Drug Injection Study Phase II differed from the phase I study in two important ways. First, it was conducted primarily in developing and transitional countries, and second, it included non-injecting drug users (both former injectors and never injectors) as well as current injecting drug users. The experience with the two studies suggests that there is much greater cross-site similarity in the drug use environments in industrialized countries and much greater cross-site diversity in drug use environments in developing and transitional countries. Thus, it was relatively easy to obtain "standardized" treatment samples in phase I cities, and very difficult to do so in many of the phase II cities. Non-injecting drug users were included in order to better understand transitions between injecting and non-injecting drug use, which are of great importance in some developing countries. Both of these factors increased the practical difficulties in conducting the study.

The fundamental tension in conducting the study was between maintaining standardized methods across the different sites and adapting to local conditions. Recruiting subjects among drug users entering drug abuse treatment is an excellent example of this issue. In cities that have a "reasonably large" treatment capacity (sufficient for 15% or more of drug users in the local area), free or low cost treatment, and voluntary entry into treatment, new entrants into treatment tend to have very high drug use frequencies and be at high risk for exposure to HIV. Thus, cross-site comparisons of new entrants into drug abuse treatment can provide important insights into the risks for HIV infection among particularly high-risk drug users in different cities. This "relatively simple" comparison could not be made for all cities in WHO phase II. For example, entry into drug abuse treatment was involuntary (reflecting law enforcement activities more than the treatment needs of the drug users) in Hanoi, Beijing, Penang, and to a lesser extent in Nairobi. In Lagos and Nairobi, although voluntary drug treatment was available, the costs were sufficiently high that only a few drug users could afford treatment. In Bogotá, where injection drug use is typically experimental and sporadic and not the primary reason for seeking treatment, the numbers of IDUs entering treatment were so small as to make recruiting from treatment entrants not only biased but impractical.

There were two basic strategies used to address the many methodological issues that arose during the study. The first was the use of rapid assessment before conducting the structured risk behavior and seroprevalence study. The rapid assessment provided critical information about how to adapt sampling strategies and questionnaire items to each local situation. This presented opportunities for resolving potential problems before they arose during the survey.

The second strategy was a close working relationship between the individual participating sites and the New York Survey Coordinating Center. The latter approved of all modifications to sampling methods and changes in the questionnaire. Any changes could be done in "real time" during the data collection and from the perspective of the study as a whole rather than having individual sites making decisions that might jeopardize the ability to pool the data across all of the sites.

## Conclusion

We certainly would not claim that the use of a rapid assessment and close coordination of a structured survey will successfully resolve all problems in attempting to maintain standardized methods across a wide variety of sites, but do believe that they are critical for achieving an acceptable level of standardized data collection across sites as diverse as those in the WHO Drug Injection Study Phase II study of HIV and injecting drug use.

## Competing interests

The author(s) declare that they have no competing interests.

## Authors' contributions

DDJ and TP coordinated the questionnaire and seroprevalence survey component of the study and provided technical support to participating sites. GS directed coordination and technical support for the rapid assessment component of the study. VP had overall responsibility for contractual arrangements with individual sites in accordance with the procedures of the WHO, and for study implementation. DDJ and TP prepared the article. All of the authors contributed substantially to the final version of the article.

## Pre-publication history

The pre-publication history for this paper can be accessed here:



## Figures and Tables

**Table 1a:** Total Recruitment

		**Africa**		**Asia**		
		Lagos	Nairobi	Beijing	Hanoi	Penang
Total		386	348	377	763	228
						
Treatment						
	Current IDUs	~	4	323	300	30
	Former IDUs	~	1^#^	6^#^	12^#^	35^#^
	Never IDUs	~	10^#^	~	9^#^	10^#^
Community						
	Current IDUs	45	102	43	345	91
	Former IDUs	29	49^#^	5^#^	39	31^#^
	Never IDUs	312	182	~	58	31^#^

~ indicates that no recruitment was attempted by design

# Originally recruited as current injectors but subsequently reclassified.

## Recruitment source unknown for 4 IDUs and 1 former IDU recruited in Rosario

**Table 1b:** Total Recruitment

		**Eastern Europe**			**South America**			
		Kharkiv	Minsk	St. Petersburg	Bogota	Gran Rosario^##^	Rio de Janeiro	Santos

Total		438	400	400	301	605	586	362
								
Treatment								
	Current IDUs	188	201	197	~	6	6	1
	Former IDUs	~	~	~	~	17	86	9
	Never IDUs	~	~	~	~	46	16	20
Community								
	Current IDUs	250	199	203	237	319	183	73
	Former IDUs	~	~	~	64	115	261	82
	Never IDUs	~	~	~	~	97	34	177

~ indicates that no recruitment was attempted by design

# Originally recruited as current injectors but subsequently reclassified.

## Recruitment source unknown for 4 IDUs and 1 former IDU recruited in Rosario

**Table 2:** Treatment Recruitment

		**Compulsory Drug Treatment**	**Voluntary Private Drug Treatment**	**Voluntary Public Drug Treatment**	**Other**
**Africa**	Nairobi	Residential drug treatment in National Psychiatric clinic^#^	Private residential drug treatment clinics		
**Asia**	Beijing	Detoxification Center			
	Hanoi	Rehabilitation center for sex workers Rehabilitation center for drug users			
	Penang	Long-term rehabilitation for drug users			

**Eastern Europe**	Kharkiv		City Detoxification Clinic	City Detoxification Clinic	
	Minsk		Minsk Narcological Dispensary	Minsk Narcological Dispensary	
	St. Petersburg			City Narcological Hospital	Psychiatric hospitals providing unofficial drug treatment

**South America**	Gran Rosario		Inpatient and outpatient therapeutic communities (typically religion-oriented) Outpatient drug clinics Drug treatment units in private psychiatric clinics and general hospital	Outpatient drug clinic Drug treatment unit in City Mental Health hospital	
	Rio de Janeiro		Outpatient drug treatment Residential drug treatment		
	Santos		Outpatient drug treatment Residential drug treatment		

# Most patients are admitted involuntarily

**Table 3a:** Demographics

		**Africa**		**Asia**		
		Lagos	Nairobi	Beijing	Hanoi	Penang

		n = 386	n = 348	n = 377	n = 763	n = 228
Female		14%	7%	23%	16%	0%
						
Age	Mean (SD)	37 (6)	29 (7)	30 (7)	28 (7)	35 (9)
	Median	36	28	30	27	33
	< 25 yrs.	2%	30%	23%	32%	8%
						
Ethnicity		71% Yoruba	68% Kikuyu	88% Han	99% Kinh	55% Malay 23%Chinese 21% Indian

**Table 3b:** Demographics

		**Eastern Europe**			**South America**			
		Kharkiv	Minsk	St. Petersburg	Bogota	Gran Rosario	Rio de Janeiro	Santos

		n = 438	n = 400	n = 400	n = 301	n = 605	n = 586	n = 362

								
Female		26%	23%	26%	21%	23%	9%	18%
Age	Mean (SD)	25 (5)	24 (5)	24 (5)	24 (7)	28 (8)	32 (10)	29(10)
	Median	24	23	23	22	27	30	27
	< 25 yrs.	55%	63%	63%	65%	43%	29%	38%
								
Ethnicity		71% Ukrainian 24% Russian	75% Belarussian 17% Russian	96% Russian	97% Mestizo	69% White 24% Mestizo	48% White 32% Mulatto 20% Black	not available
